# Adding Color to Mass Spectra of Biopolymers: Charge Determination Analysis (CHARDA) Assigns Charge State to Every Ion Peak

**DOI:** 10.1021/jasms.3c00442

**Published:** 2024-04-12

**Authors:** Yaroslav Lyutvinskiy, Konstantin O. Nagornov, Anton N. Kozhinov, Natalia Gasilova, Laure Menin, Zhaowei Meng, Xuepei Zhang, Amir Ata Saei, Tingting Fu, Julia Chamot-Rooke, Yury O. Tsybin, Alexander Makarov, Roman A. Zubarev

**Affiliations:** † Division of Chemistry I, Department of Medical Biochemistry and Biophysics, Karolinska Institutet, SE-17 177 Stockholm, Sweden; ‡ Spectroswiss, 1015 Lausanne, Switzerland; § Ecole Polytechnique Fédérale de Lausanne, 1015 Lausanne, Switzerland; ∥ Department of Cell Biology, Harvard Medical School, Boston, Massachusetts 02115, United States; ⊥ Biozentrum, University of Basel, 4056 Basel, Switzerland; # Centre for Translational Microbiome Research, Department of Microbiology, Tumor and Cell Biology, Karolinska Institutet, Stockholm 17165, Sweden; ∇ Institute Pasteur, 75015 Paris, France; ○ ThermoFisher Scientific, 28199 Bremen, Germany; □ Department of Pharmacological & Technological Chemistry, I.M., Sechenov First Moscow State Medical University, 119991 Moscow, Russia; ◊ The National Medical Research Center for Endocrinology, 115478 Moscow, Russia

**Keywords:** mass spectrometry, polypeptide, proteomics, spectrum

## Abstract

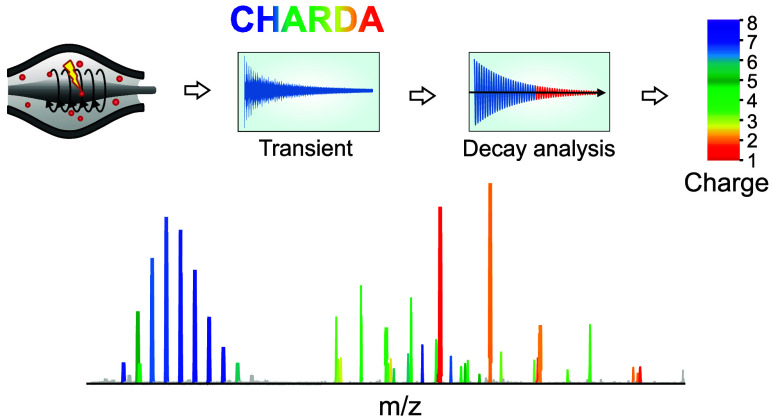

Traditionally, mass spectrometry (MS) output is the ion abundance plotted versus the ionic mass-to-charge ratio *m*/*z*. While employing only commercially available equipment, Charge Determination Analysis (CHARDA) adds a third dimension to MS, estimating for individual peaks their charge states *z* starting from *z* = 1 and color coding *z* in *m*/*z* spectra. CHARDA combines the analysis of ion signal decay rates in the time-domain data (transients) in Fourier transform (FT) MS with the interrogation of mass defects (fractional mass) of biopolymers. Being applied to individual isotopic peaks in a complex protein tandem (MS/MS) data set, CHARDA aids peptide mass spectra interpretation by facilitating charge-state deconvolution of large ionic species in crowded regions, estimating *z* even in the absence of an isotopic distribution (e.g., for monoisotopic mass spectra). CHARDA is fast, robust, and consistent with conventional FTMS and FTMS/MS data acquisition procedures. An effective charge-state resolution *R*_*z*_ ≥ 6 is obtained with the potential for further improvements.

## Introduction

Traditionally, mass spectrometry (MS) measures the mass-to-charge ratio *m*/*z* of ions, providing two-dimensional mass spectra where the ion abundance is plotted against the ionic *m*/*z*. At the same time, the main parameters of interest are the compound’s neutral mass *m* and abundance. During the first seven decades of MS development, the absolute majority of mass spectra contained only singly charged ions, and with the experimental mass error being much larger than the electron mass, the *m*/*z* values were, in practice, equal to the neutral mass. The situation has changed with the advent of electrospray ionization (ESI) and similar techniques producing multiply charged ions of polypeptides.^1^ In response, various approaches have emerged aimed at estimating *m* from multicharge MS data. Up to now, the two main strategies of charge-state deconvolution have both been based on using several ion peaks originating from the same molecule. In lower resolution mass spectra of biopolymers, the *m*/*z* difference between different charge states is employed.^2^ In high-resolution mass spectra, where individual isotopic peaks are resolved, *z* can be deduced as the closest integer to (1.003*k*)/Δ*m*/*z*, where Δ*m*/*z* is the distance on the *m*/*z* scale between the *N*th and (*N* + *k*)th peaks in an isotopic cluster, with *N* being a positive integer. However, in crowded mass spectra, the isotopic clusters often overlap, making it difficult to determine which of the many closely spaced ion peaks belong to the same isotopic cluster.^3^

To address this issue, several methods have emerged to estimate the charge states of individual ion peaks independently from the other ions in a mass spectrum. Most of these methods are based on measuring the charge of individual ions. An early approach used a secondary ion detector in which the number of secondary ions created by an incident analyte ion depended upon the ion kinetic energy. As the kinetic energy of an ion accelerated by a potential difference *ΔU* is *zΔU*, the number of secondary ions depends upon the ionic charge *z.*(4) Similarly, a time-of-flight cryodetector could measure the kinetic energy of the ions impacting the detector, which correlated with the ionic charge.^5^ Charge detection mass spectrometry (CDMS) measures the charge of individual ions by the signal induced by them on an image current detector that records a time-domain signal or transient. CDMS then applies the Fourier transform (FT) or a similar approach to these time-domain signals to derive *z* and *m*/*z* of ions.^6^ A recent technique called individual ion MS (I^2^MS) implements CDMS in commercially available Orbitrap mass spectrometers.^7^ In the I^2^MS approach, ≤120 individual ions are obtained per spectrum acquisition with thousands of acquisitions obtained to generate a full mass spectrum. The ionic charge state is estimated based on the fact that the ion peak’s abundance increases with time-domain transient duration in a charge-state-dependent manner. I^2^MS can assign the charge state even in the absence of isotopic resolution, which is particularly useful for very large ions, such as protein complexes. On the other hand, many thousands of acquisitions may be required to generate the charge calibration curve by the I^2^MS approach, and it is currently limited by the lowest charge state *z* ≈ 10 on commercial instruments. Increasing time-domain transient duration in I^2^MS improves the charge-state limit of detection,^8^ albeit at the expense of a proportionally increased experimental time. The latter also require performance improvements of the data acquisition system employed for the extended-length transient acquisition and processing. In measuring *z* = 22+ ions, full width at half-maximum (fwhm) charge-state resolution *R*_*z*_ ≈ 2 has been obtained,^7^ with higher charge resolution for higher charge states.

Here, we present the Charge Determination Analysis (CHARDA) method that is applicable in FTMS for all charge states starting from *z* = 1. In CHARDA, a calibration curve can be obtained from a single mass spectrum acquired with commercially available FTMS equipment in a conventional data acquisition mode, allowing for postprocessing of already acquired MS and MS/MS data (time-domain transients). While not requiring the detection of single (individual) ions or a very small number of ions per mass spectrum, CHARDA still adds a third dimension to MS by estimating the charge state *z* for every ion peak in the FT mass spectrum. For the compact representation of 3D information, the ion charge state is displayed by color coding the ion peaks.

## Experimental Section

### Sample Preparation

Solvents, including water, acetonitrile (ACN), and formic acid (FA), were employed at LC-MS purity grade. Water and ACN were purchased from Fluka Analytical (Buchs, Switzerland). FA was obtained from Merck (Zug, Switzerland). Standard proteins, namely, bovine insulin, myoglobin, and carbonic anhydrase, were obtained from Sigma-Aldrich (Buchs, Switzerland). Sample preparation protocols were identical with the standard ones described elsewhere.^9^ Infliximab sample was obtained from Sigma. *E. coli* bacteria were grown in the normal and isotopically depleted (for ^13^C, ^2^H, ^15^N, and ^18^O) media.

### Mass Spectrometry

Two Q Exactive HF Orbitrap FT mass spectrometers (Thermo Fisher Scientific, Bremen, Germany) installed in different laboratories were interfaced with high-performance data acquisition and processing (DAQ/P) systems (FTMS Booster X2, Spectroswiss, Lausanne, Switzerland) as described elsewhere.^9^ The Orbitrap was operated via standard instrument control software (Tune 2.9 and Xcalibur 4.1, Thermo Fisher Scientific). Full MS or tandem MS (MS/MS) data were acquired in positive-ion mode using ESI with the standard settings. Ion population inside the mass analyzer was controlled via the automatic gain control (AGC) capability. The instrument was operated with standard resolution settings of 15 000–240 000 at *m*/*z* 200 (in enhanced FT, or eFT, mode) which correspond to ion detection periods (transient lengths) of 32–512 ms. The Orbitraps were externally calibrated using the regular calibration mixture (composed of caffeine, MRFA, and Ultramark). The time-domain transients were acquired in parallel with the eFT mass spectra with the FTMS Booster X2 interfaced to the Orbitrap instrument via standard digital and analog output connectors. Acquisition of extended length transients (1.0–1.5 s) was enabled by the parallel ion detection and accumulation capability of the Orbitrap and flexible triggering capabilities of the FTMS Booster X2, as described elsewhere.^10,11^ Briefly, dummy scans with 1.0–1.5 s ion accumulation time (ITmax) were introduced between the analyte scans to extend the period of analyte ion trapping (oscillation) in the Orbitrap mass analyzer. The thus acquired time-domain transients were preprocessed with Peak-by-Peak software (Spectroswiss) and provided for further data processing and analysis using the dedicated software tools developed in this work.

The experimental results described in this paper stem from the following four data sets: (i) higher energy collision-induced dissociation (HCD) MS/MS analysis of the isolated 11+ charge state of ubiquitin (AGC setting of 1e6, 20 *m*/*z* isolation window, 350 individual time-domain transients of 1.5 s duration each); (ii) MS-only mass measurements of a protein mixture that included myoglobin and carbonic anhydrase in different charge states performed in the denaturing conditions (1 s time-domain transients, AGC settings were varied between 1e6 and 5e6); (iii) MS/MS analysis of a monoclonal, antibody sample, Infliximab; and (iii) *E. coli* tryptic digest (1.6 s time-domain transients). The *E. coli* data set included analysis of the isotopically normal and isotopically depleted samples. The LC-MS measurements were performed using a C4 column (Waters), and the AGC was set at 3e6.

## Results

### Algorithm Description

**Figure 1:**
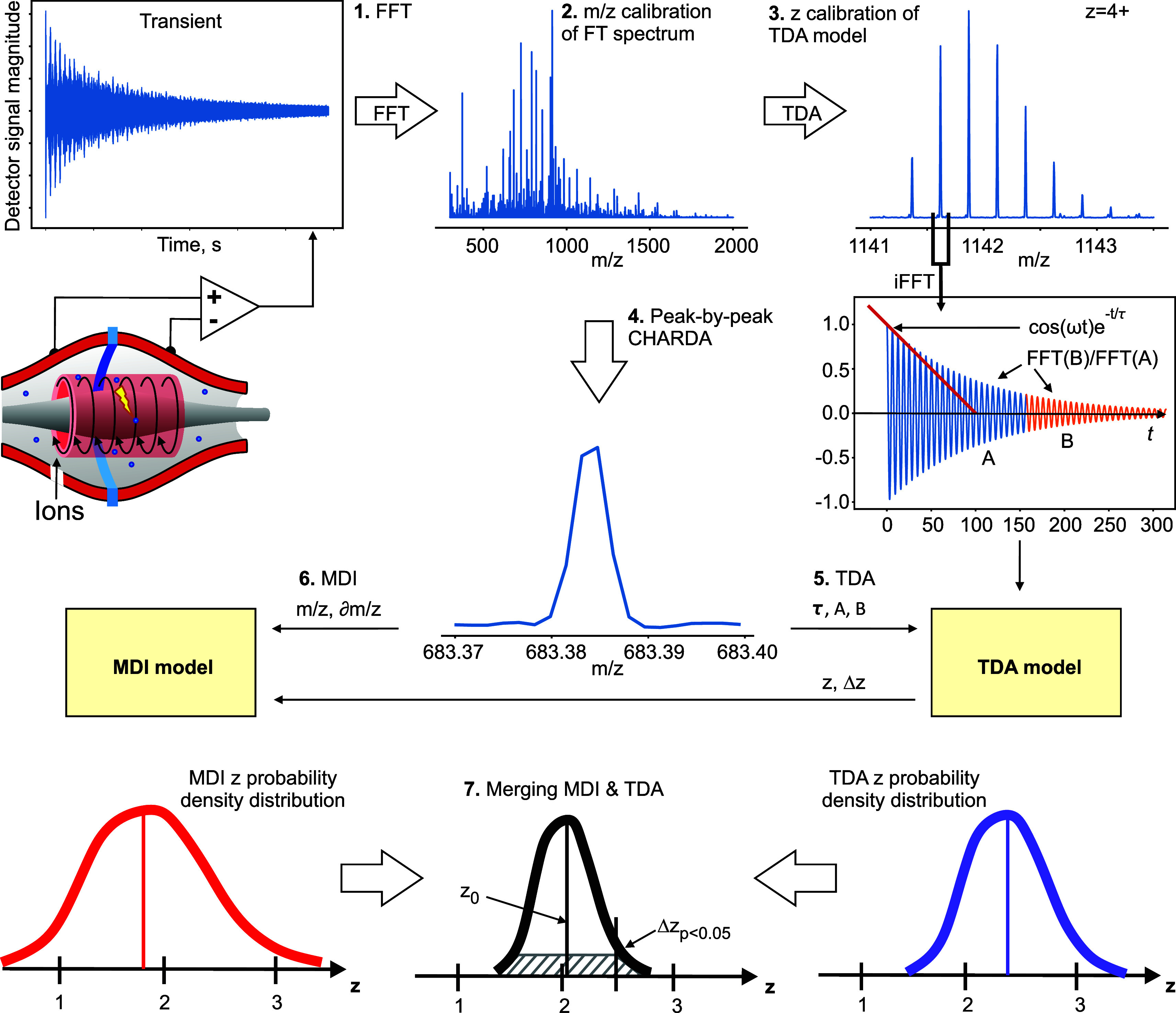
Charge determination analysis (CHARDA). (1) In transient decay analysis (TDA), the time-domain signal (transient) obtained from a FT mass analyzer is converted by FFT to a frequency spectrum. (2) The latter is converted to an *m*/*z*-scale spectrum using known ion peaks or external calibration. (3) Narrow *m*/*z* areas around individual peaks are converted to time-domain transient using inverse FFT. The TDA model is calibrated with parameters of the transients and charge states of the known ions. (4) Peak-by-peak TDA of the whole mass spectrum is performed, and for each peak, a charge-state probability density distribution is obtained. (5) This distribution is then transferred to mass defect interrogation (MDI). (6) MDI provides its own charge-state probability density distribution. (7) The TDA and MDI charge-state probability density distributions are merged, with the most probable charge state *z*_0_ and the charge interval *Δz*_*p<0.05*_ corresponding to *p* < 0.05 determined.

CHARDA combines the complementary approaches of time-domain transient decay analysis (TDA) characteristic for FTMS with mass defect (fractional mass) interrogation (MDI) that can be implemented using any high-resolution mass spectra (Figure 1). TDA utilizes the well-known fact that in FTMS the time-domain transient, theoretically being nearly sinusoidal, is actually more complex, often close to a sinusoidal wave with exponentially decaying amplitude (Figure 1, upper left). In the high-pressure limit, the decay is attributed to collisions with the residual gas.^12,13^ The exponent parameter known as the decay constant 1/τ is thus linked to the collisional cross section (CCS).^12−14^ For ions with a similar *m*/*z*, a larger mass (and thus also higher charge state) tends to imply larger CCS and thus faster transient decay. Indeed, the ions in the *m*/*z* interval 772–780 have the following experimental CCS values: ubiquitin 11+ (8.6 kDa) – 2394 ± 21 Å^2^, apomyoglobin 22+ (17.0 kDa) – 4887 ± 74 Å^2.^^15^ In ion mobility MS, very similar CCS values (2340 and 4920 Å^2^, respectively) have been obtained for these ions.^16^ Thus, we concluded that decay constants in FTMS must carry charge-predictive information. Indeed, using the linear regression of the above values for 11+ ubiquitin and 22+ apomyoglobin, the CCS of 3529 ± 99 Å^2^ corresponds to the charge state *z* = 16.004 ± 0.010, a very accurate estimate for the 16+ cytochrome *C* ions with such an experimental CCS value determined by FTMS (the charge state was estimated as (3529 – 2394)/(4887 – 2394) × (22 – 11) + 11).^15^

Note that for the charge state, which is a discrete value, the estimation accuracy in the above example is greatly excessive. Monte Carlo simulations showed that as long as the standard deviation of both calibration CCSs is kept below 2.4%, more than 95% of the charge-state estimates approximated to the nearest integer were correct, i.e., 16+. With a ≤6.7% standard deviation, >95% of cases gave the correct charge state within a ±1 charge-state margin, and with a ≤10.6% standard deviation it was within ±2 charge-state units. Even such approximate charge-state assignment can be useful in practice.

In TDA, the transient is first converted using the fast Fourier transform (FFT) to a frequency domain spectrum and the latter to an *m*/*z* spectrum (Figure 1 (1)). The ion peaks are distinguished from the background by an appropriate algorithm. Narrow *m*/*z* areas around every individual peak are then converted to a time-domain partial transient using inverse FFT, and the decay parameter of this partial transient is estimated. Even though the normal Orbitrap operation is not within the high-pressure limit (typical pressure being ∼10^–10^ mbar), TDA assumes that transient decay is largely caused by collisions with neutral gas. There are several known ways to estimate the decay constant 1/τ, including a selective temporal overview of resonant ions (STORI) plots^17^ and finite impulse response (FIR) filtering,^18^ but we have found experimentally that fitting a decaying exponent exp(−*t*/τ) to the time interval of 0.2–1.0 s (more general, from 1/8 to 5/8 of the transient) provides the best charge-state resolution for the data under consideration.

In parallel, FFT of the two halves of the full transient, A and B, is performed, each half-transient providing an ion peak at the same position as the full transient but at one-half the *m*/*z* resolution. The ratio of the intensities of the peak in FFT(B) versus FFT(A) is  (Figure 1 (3)); the decay constant 1/τ and the peak *m*/*z* and abundances are then combined in a single *z-*predictive TDA model.

This model is calibrated as follows. First, a conventional algorithm of charge-state deconvolution based on the isotopic distribution of ions is applied to the whole *m*/*z* spectrum, generating a list of identified isotopic clusters with reliably determined charge states. These usually encompass <20% of the ion peaks in a mass spectrum. Then, a logistic regression model is built based on these reliably identified charge states predicting *z* from the transient parameters for any ion peak.

The position of the ions on the *m*/*z* scale also carries information about the molecular mass and thus about the charge states of ions *z*. A large fraction of all FTMS analyses performed to date concerns polypeptides, and thus, our discussion will be limited to these biopolymers. The mass defect interrogation (MDI) investigates the ionic mass defect *d*_*m*_, which is defined here as a difference between the accurate neutral mass of a compound and its nearest lower mass integer approximation, e.g., mass defect of *m* = 2083.8753 Da is *d*_*m*_ = 0.8753 Da. For monoisotopic polypeptide molecular masses, the mass defect is a periodic function with a period of 1.9–2.2 kDa.^19−22^ MDI uses the well-known fact that the monoisotopic molecular mass defects are not evenly distributed over the mass scale but are clustered around their average values.^19^ For instance, for the monoisotopic polypeptide masses between 1000 and 1001 Da, the mass defect distribution is centered around 0.55 Da with a majority of molecules falling within the ±0.1 Da interval around this value.^21^ For other isotopic peaks of proteins, mass defects increase by ∼3 mDa for every Dalton away from the monoisotopic peak. This is because the ^13^C is the main contributor to the isotopic distribution with the mass difference between ^13^C and ^12^C being ∼1.0033 Da, while the presence of other elements makes the average interisotope distance in polypeptides closer to 1.003 Da.

Since the difference between the average isotopic mass and the monoisotopic one increases by 1 Da for every 1.6–1.8 kDa of molecular mass increase^19^ and the mass of the most abundant isotopologue is always ≤1 Da below the average isotopic mass,^23^ for polypeptides below 25 kDa, the most abundant isotopologue has a mass defect within 0.05 Da of the monoisotopic mass defect (disregarding the discontinuity occurring when *d*_*m*_ reaches 1.0). Therefore, knowing the theoretical distribution of mass defects as a function of polypeptide mass, one can obtain a probability estimate for different charge states of that mass. As an example, for a polypeptide ion with *m*/*z* 1000.200, the charge state 1+ is highly unlikely as the mass defect of 200 mDa falls within the forbidden “band gap” between the mass distribution peaks. According to Mann,^20^ the molecular mass at which the interval encompassing ≥95% of all amino acid compositions exceeds 1 Da is ∼8 kDa. Thus, around and beyond that critical mass the band gap disappears and the distribution of monoisotopic protein masses becomes pseudocontinuous (there are still gaps on a microscale). However, the differences between the regular maxima and minima of the distribution remain significant for much larger masses, and thus, MDI analysis is still plausible.

Molecular mass defects have been previously used to estimate the probability of a given mass to belong to a peptide^24^ or to a modified peptide.^25^ But, the use of *d*_*m*_ to augment charge-state determination in polypeptides appears to be novel,^26^ while the link between the mass defect and the charge state for synthetic polymers has recently been explored.^27^ In our implementation, MDI uses not only monoisotopic molecular masses but also polyisotopic masses as the charge state is predicted for any ion peak regardless of its position in the isotopic cluster.

When CHARDA is applied to an individual *m*/*z* peak resolved in a mass spectrum (Figure 1 (4)), both the TDA model and the MDI model predict the ion’s charge state and its probability density distribution (Figure 1 (5 and 6)). The MDI model is universal and only needs to be adjusted if a different class of molecules than unmodified polypeptides is considered (e.g., specifically modified polypeptides, polymers, RNAs, sugars, etc.). To create such a model from scratch, a theoretical mass distribution needs to be simulated for a given class of molecules, including not only monoisotopic masses but also those of other isotopologues. The discrete nature of this distribution^28^ can cause inconveniences, and thus, the distribution has to be averaged with a narrow window (e.g., 0.01 Da), providing smooth periodic envelopes. The envelopes have distinct peaks at low molecular masses with “band gaps” between them. The abundance of a peak at each mass unit is normalized so that its area is unity. Such normalization converts the mass distribution into a mass-dependent probability distribution function. The probability of a certain discrete *z* for a specific *m*/*z* value is established, assuming that the ionic charge is due to protonation. Therefore, the value of (*m*/*z* – *m*_*p*_)·*z* with its error band (*m*_*p*_ = 1.007277 is the proton’s *m*/*z*) is mapped onto the probability distribution, and the area under the curve (AUC) is established. In a similar manner, AUCs are obtained for other plausible *z* values, and all thus obtained AUCs are normalized such that their sum is unity. In the MDI model, the normalized AUCs represent the probabilities for a given *m*/*z* to correspond to respective *z* values.

The TDA and MDI probability charge-state density distributions for a given ion peak are merged, giving rise to a single distribution (Figure 1 (7)). The most probable charge-state value (not necessarily an integer) as well as the interval of charge-state values corresponding to >95% certainty (*p* < 0.05) are thus determined. This charge-state value is then color coded into the ion peak envelope in the mass spectrum, providing a third dimension of information.

The source code implementing CHARDA is provided as a CodeOcean capsule with no restrictions. The same capsule contains all necessary data and source code for the application of CHARDA to form the article figures.

### Proof of Principle

**Figure 2:**
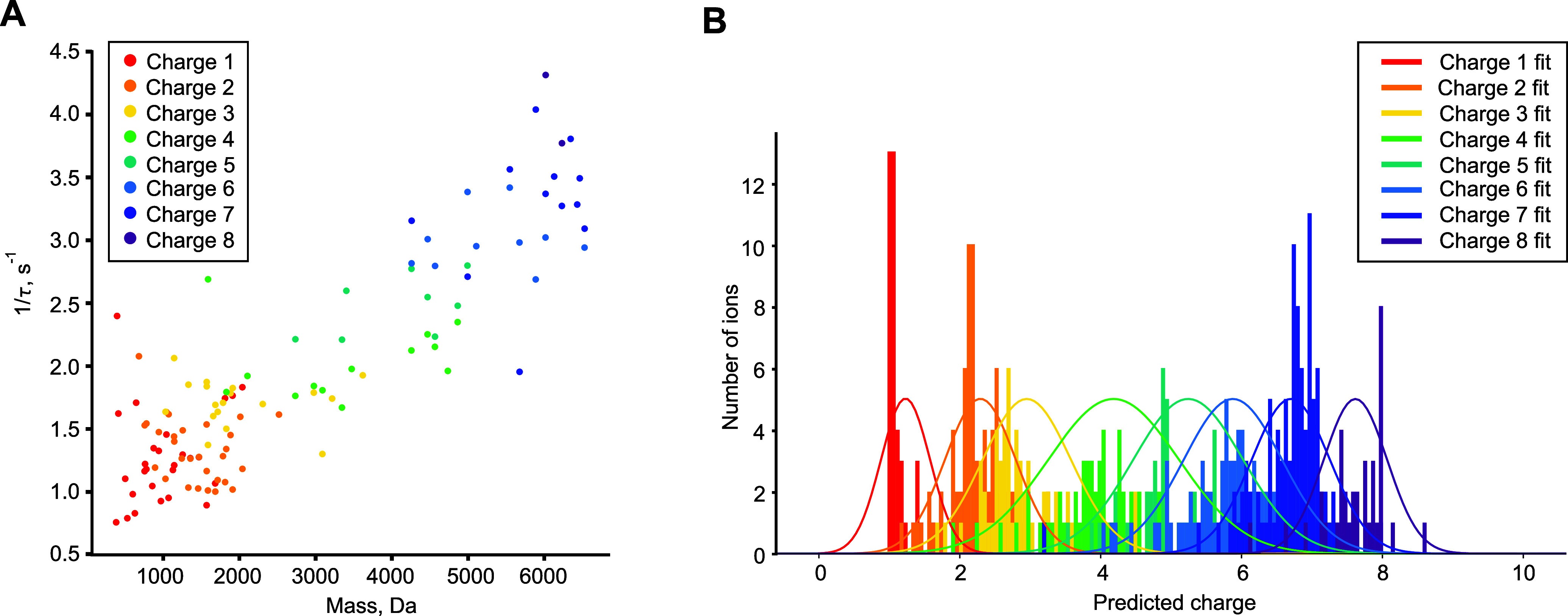
TDA proof of principle. (A) TDA-extracted decay constants of the calibration ions of ubiquitin 11+ fragments versus their molecular mass. (B) The distribution of the CHARDA-predicted charge states of the calibration peaks and their Gaussian fits versus the charge determined by the deconvolution algorithm (Hardklör).

As a proof of principle, we analyzed an MS/MS spectrum of ubiquitin 11+ ions acquired on a Q Exactive HF Orbitrap (Thermo Fisher Scientific) as a sum of 350 individual transients, each with a 1.5 s duration.^29^ The mass spectrum contains 33 796 ion peaks, of which 7208 peaks (21% of the total) representing 2323 isotopic clusters were charge-state deconvolved using the Hardklör algorithm.^30^ In total, 1167 peaks in 212 clusters were recognized as being due to ubiquitin *b/y* product ions, and 533 were the most reliable of them, representing 159 isotopic clusters with *z* ranging from 1+ to 9+. These were used for the TDA model calibration, Figure 2. Figure 2A demonstrates that the TDA-extracted decay constants scale nearly linearly with the monoisotopic molecular mass of the calibration ions. Figure 2B shows the distribution of the CHARDA-predicted charge states of these peaks and their Gaussian fits versus the charge state determined by the Hardklör algorithm.

In order to optimize the CHARDA algorithm, a quantitative measure of its performance was needed. Such a measure was charge-state resolution determined as the maximum value of the predicted charge state divided by the fwhm of the fitted bell curves as in Figure 2B averaged over all charge states. The thus determined TDA resolution was 5.6, while the addition of MDI improved the resolution to 6.0 (Figure S1, Supporting Information). As expected, most of the improvements were observed for the lower charge states, starting from *z* = 1.

**Figure 3:**
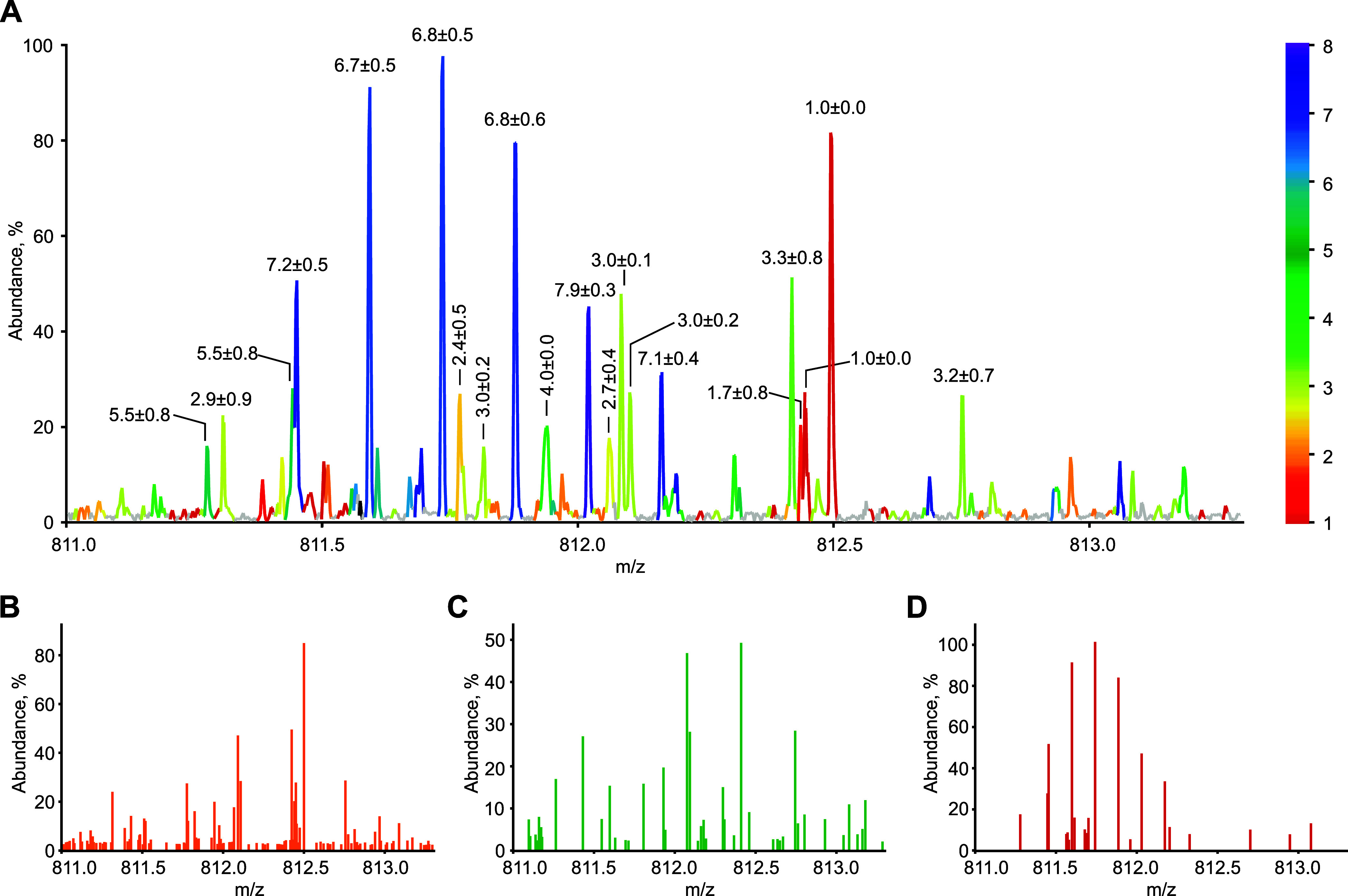
CHARDA of a convoluted MS/MS spectrum region. Charge prediction for individual ion peaks (A). Subsets of ion peaks with predicted charges *z* ≤ 4 (B), 3 ≤ *z* ≤ 6 (C), and *z* ≥ 5 (D).

Figure 3 shows the application of CHARDA to a convoluted region of the MS/MS spectrum. Figure 3A shows a spectral region between *m*/*z* 811.0 and 813.5. The color-coded peaks allow one to easily discern separate isotopic clusters. To reduce the complexity, the spectrum can be divided into three subspectra of overlapping charge-state intervals, e.g., *z* ≤ 4, 3 ≤ *z* ≤ 6, and *z* ≥ 5 (Figure 3B–D).

Hardklör deconvolution of the whole MS/MS spectrum presented in an expanded view in Figure 3 gave 212 isotopic clusters. To estimate the effect of CHARDA on isotopic deconvolution, the entire MS/MS spectrum was divided into eight subspectra, one for every predicted charge state, with peaks having a >1% probability of being in a given charge state included in the corresponding subspectrum. In each subspectrum, Hardklör deconvolved the isotopic clusters, producing 19 additional isotopic clusters (a 9% improvement) that were missed in the full-spectrum deconvolution. The masses of all of these clusters corresponded to expected fragment ions of ubiquitin. This result confirms that CHARDA can improve isotopic cluster deconvolution for complex tandem mass spectra.

To test whether CHARDA can be implemented in a different laboratory (the original data reported above were collected at the Ecole Polytechnique Fédérale de Lausanne), we implemented CHARDA in Institute Pasteur on the analysis of the monoclonal antibody Infliximab (Figure S2).

### Manual Mass Spectra Inspection

**Figure 4:**
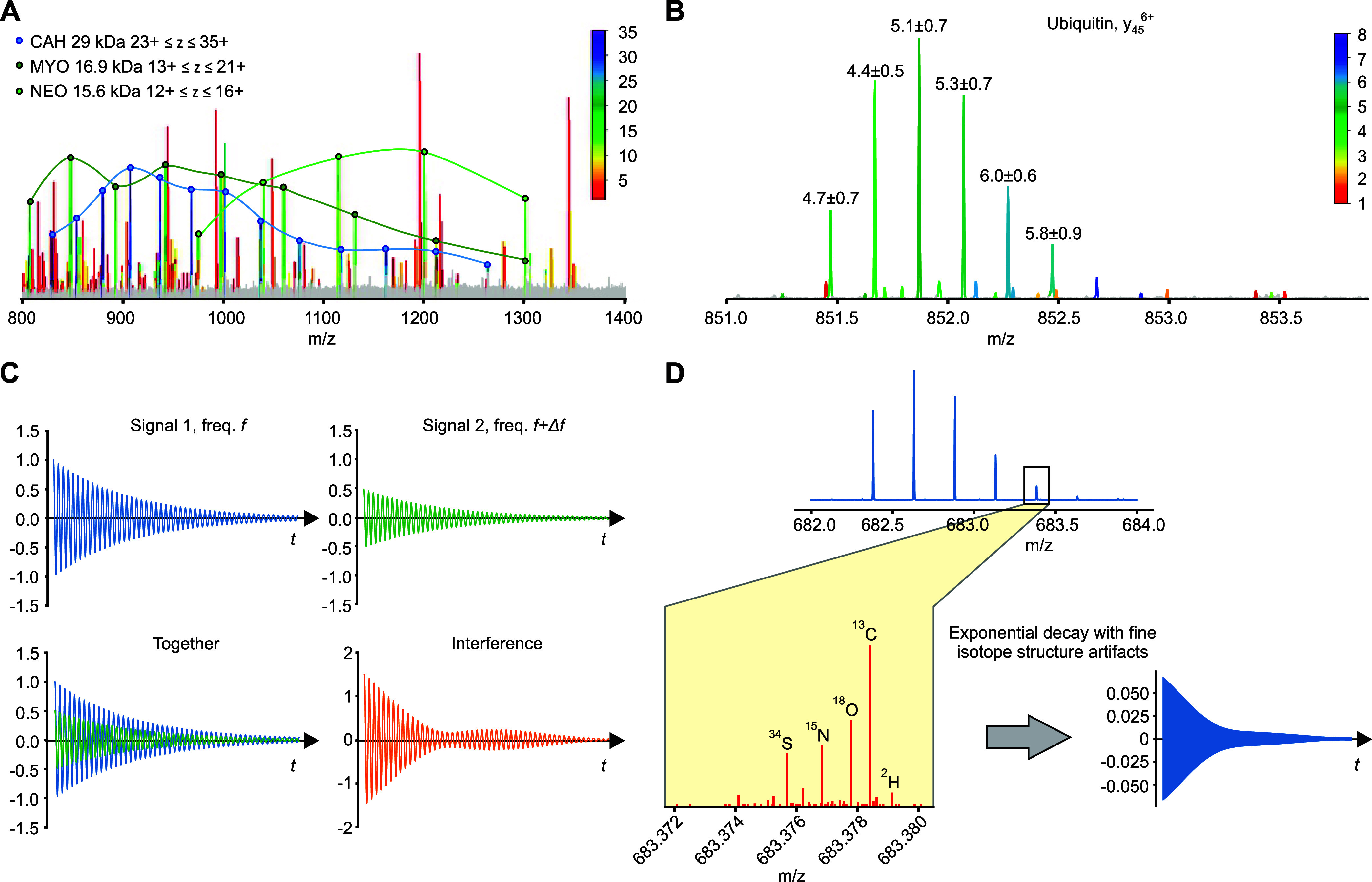
CHARDA implementation and features. (A) Manual inspection of convoluted mass spectra of polypeptide mixtures can be facilitated by CHARDA color coding, with charge ladders connected by lines for better visibility. CAH, carbonic anhydrase; MYO, myoglobin; NEO, unidentified protein, possibly a hydrolysis product. (B) A monotonous increase of the CHARDA-predicted charge state for high-order isotopologues (ion peaks more distant from the monoisotopic peak). (C) An interference of two close frequencies with equally decaying amplitude gives a pattern with a complex amplitude envelope. (D) The isotopic distribution of the *y*_24_ ubiquitin fragment and the calculated isotopic fine structure of its 5th isotopologue.

Another possible CHARDA application is the manual inspection of mass spectra with overlapping charge-state distributions of molecular or fragment ions. This is particularly useful when proteins exist in different proteoforms,^31^ and thus, there is no clear “charge-state ladder” in mass spectra, as exemplified by Figure 4. Figure 4A shows a color-coded mass spectrum with a 1 s transient duration of a mixture of three proteins. Color coding was made by TDA without accurate calibration of charge states by instructing the algorithm to recognize different charge states using the maximum likelihood approach. For clarity, the charge-state ladders are connected by lines. While many peaks obviously do not belong to any ladder, their tentative attribution to this or that protein molecule can be facilitated by peak color coding. This attribution can be verified by other approaches, such as charge-state deconvolution by Hardklör or MS/MS.

### Benefit of Isotopic Resolution

Without isotopic resolution, the ion peak represents a weighted average of the whole isotopic envelope. We performed numerous CHARDA attempts on transients shortened from 1 to 0.5 s that gave isotopic distributions merged into a single peak but were unable to predict charge states correctly at such conditions. Thus, unlike I^2^MS, CHARDA requires high mass resolution (i.e., mass resolution at which the isotopic cluster is resolved into individual peaks) but does not require the actual presence of other isotopic peaks for charge estimation performed on one such peak.

### Charge State of Individual Isotopologues

Close inspection of the CHARDA-predicted *z* values for individual isotopologues revealed a systematic increase with the isotopologue distance from the monoisotopic peak (see as an example the ubiquitin *y*_45_^6+^ ion in Figure 4B). To verify this effect, we split the isotopic distributions of the peaks with *z* = 5+, 6+, and 7+ with similar signal-to-noise ratios into the left and right halves (Figure S3a) and measured the average decay rate for the ion peaks in each half separately. For all three charge states, the right (heavier) parts showed significantly faster decay (Figure S3b). The origin of this effect could be the increased multiplicity of the isotopic fine structure (IFS) for peaks more distant from the monoisotopic one.^32^ Indeed, a mixture of two close frequencies with equally decaying amplitude gives a pattern with a complex amplitude envelope (Figure 4C) from which the extraction of the original decay constant is nontrivial.^33^ We calculated the IFS of the fifth isotopic peak of the *y*_24_ ubiquitin fragment and simulated its transient as a weighted sum of the equally decaying exponents of the individual IFS components (Figure 4D). The TDA of that summed transient gave a 47% larger decay constant than that of each component. As an effective compensation method for this artifact has not been found so far,^33^ we hypothesized that CHARDA should work best for molecules with dominant monoisotopic masses.

Another potential limitation of the charge resolution in CHARDA is the regularity of scaling the CCS values. In other words, the error of the estimated charge state increases when the ions represent a mixture of collapsed and extended molecules. For instance, we noted that in terms of charge deconvolution, *y* and *b* ion series have more in common among themselves than with the opposite series. The charge deconvolution algorithm would also be confused if multiple charge states overlapped perfectly, such as in a dimer on top of a monomer with one-half the charge.

### MS/MS of Monoisotopic Proteins

**Figure 5:**
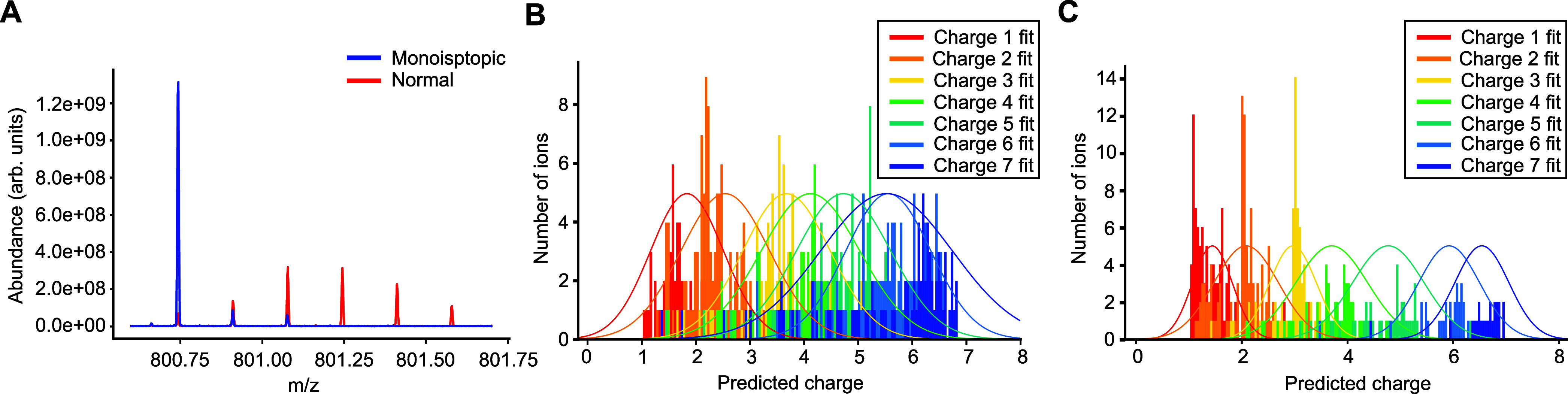
Comparison of CHARDA performance in top-down analysis of *E. coli* bacteria grown in the normal and isotopically depleted (monoisotopic) media. (A) An example of a 6+ molecular ion. (B) Performance of CHARDA (TDA + MDA) models for MS/MS fragments of 5 different proteins for the full isotope data set, *R* = 3.4. (C) Performance of CHARDA (TDA + MDA) models for MS/MS fragments of 5 different proteins for the monoisotopic data set, *R* = 5.4.

Proteins are large molecules with an average size of bacterial proteins > 30 kDa, while the monoisotopic ion peak is usually indiscernible in the noise at MW > 10 kDa. Thus, to create proteins with abundant monoisotopic ion peaks and test CHARDA applicability on them, we grew *E. coli* bacteria in minimal media with 3–30-fold-depleted ^2^H, ^13^C, ^15^N, and ^18^O isotopes as well as in normal media for control. At the same amount of protein loaded, LC-MS of the soluble proteome gave 3–5 times more abundant signal for the depleted proteins, as the signal concentrated in fewer isotopic peaks (Figures 5A and S4). This resulted in 283 proteoforms selected for MS/MS in depleted proteome versus 120 proteoforms for normal proteome. Hardklör could not process depleted proteome data in the automatic mode and had to be supplemented with manual analysis. MS/MS identified at least 9 proteoforms in both normal and monoisotopic data sets, and 5 proteoforms with 413 monoisotopic peaks in total were used for CHARDA calibration. Consistent with our expectations, CHARDA provided higher resolution (Figure 5B and 5C), namely, *R* ≈ 5.4 with both TDA and MDI and *R* ≈ 4.5 with TDA only for the monoisotopic proteome versus *R* ≈ 3.2 and 3.4, respectively, for the normal proteome.

After calibration, CHARDA could easily identify the charge states of ions for which conventional deconvolution algorithms provided no results. In total, CHARDA identified 1120 backbone fragments of five proteins and estimated charge states for them in the monoisotopic MS/MS data set, providing on average 81% sequence coverage (Table S1). Of these, only 321 fragments were identified by Hardklör in the corresponding full-isotope MS/MS data set, giving, on average, 41% sequence coverage. The performance of the CHARDA models for the normal (656 peaks) and monoisotopic (413 peaks) MS/MS data are presented in Figure 5B and 5C, providing *R* = 3.4 for the normal case and *R* = 5.4 for the monoisotopic case.

### Simplified CHARDA

**Figure 6:**
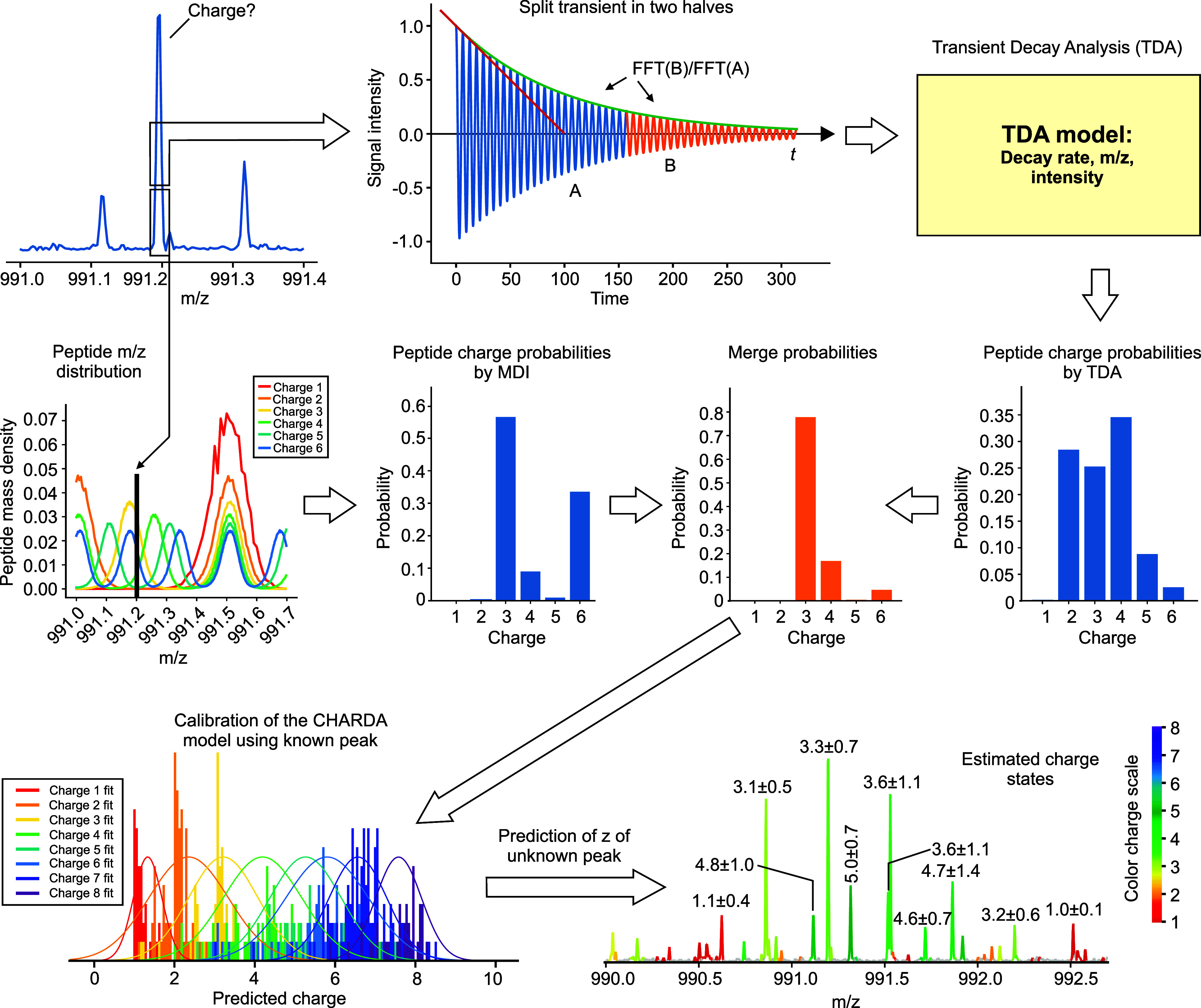
A simplified CHARDA (sCHARDA) version. Compared to the standard FTMS spectrum processing, sCHARDA adds only FTs of the two half-transients to the normal FT procedure, which maximum doubles the FT calculation time.

Full-scale CHARDA analysis requires inverse FT of hundreds and thousands of individual peaks, which is calculation intensive. Thus, we tested the simplified CHARDA version (sCHARDA, Figure 6) which, compared to the normal FTMS spectrum processing, adds to the normal FT procedure only FTs of the two half-transients, maximum doubling the FT calculation time. Therefore, the inverse FT step is omitted. Yet, the charge-state resolution obtained with sCHARDA is comparable with that of the full-scale CHARDA (*R* ≈ 4.0 for ubiquitin 11+ MS/MS spectrum in Figure 3), which is sufficient in the majority of cases.

## Conclusions

Here, we introduced a novel data acquisition and processing method, CHARDA, to help perform structural analysis of biopolymers with MS and MS/MS. We demonstrated that CHARDA can determine the charge states of separate ion peaks at the conditions of isotopic resolution even when the isotopic distribution is actually absent due to isotope depletion.^7^ Unlike other methods,^7^ CHARDA has no limitation on the lowest charge state and requires no special data acquisition technique except for transient recording. The color coding of the ionic charge states resulting from CHARDA provides a third dimension to mass spectra, facilitating the analysis of crowded MS and MS/MS data and hinting at possible peak interferences and overlaps. The simplified CHARDA version, sCHARDA, can be implemented in real time, online with data acquisition on most FTMS instruments.

## Data Availability

The source code of CHARDA is available from https://github.com/Yaroslav-Lyutvinskiy/CHARDA under Apache 2.0 license agreement. All data and computations presented in the current article are also available from the same repository.

## Supporting Information Available

Comparison of top-down LC-MS/MS analysis of *E. coli* proteome with normal isotopic composition (full isotope) and isotopically depleted (monoisotopic); CHARDA (TDA + MDI) model based on ubiquitin 11+ MS/MS data; CHARDA of an MS/MS spectrum of monoclonal antibody Infliximab; example of splitting the isotopic distribution of the *y*_37_^6+^ ubiquitin ions and decay rate comparison (PDF)
