# Supplementary material for: Adding Color to Mass Spectra of Biopolymers: Charge Determination Analysis (CHARDA) Assigns Charge State to Every Ion Peak
Source: J Am Soc Mass Spectrom. 2024 Apr 12;35(5):902–11. doi: 10.1021/jasms.3c00442 (PMC11066971; doi:10.1021/jasms.3c00442)
Supplement: Supplementary file 1 — js3c00442_si_001.pdf [file js3c00442_si_001.pdf]

Supporting Information

Adding Colour to Mass Spectra of Biopolymers: Charge Determination Analysis (CHARDA) Assigns Charge State to Every Ion Peak

Yaroslav Lyutvinskiy,[1] Konstantin O. Nagornov,[2] Anton N. Kozhinov,[2] Natalia Gasilova,[3] Laure Menin,[3] Zhaowei Meng,[1] Xuepei Zhang, [1] Amir Ata Saei,[1,4,5,6] Tingting Fu, [7] Julia Chamot-Rooke, [7] Yury O. Tsybin,[2] Alexander Makarov,[8] Roman A. Zubarev[1,9,10]*

[1]Division of Chemistry I, Department of Medical Biochemistry and Biophysics, Karolinska Institutet, SE-17 177 Stockholm, Sweden
[2]Spectroswiss, 1015 Lausanne, Switzerland
[3]Ecole Polytechnique Fédérale de Lausanne, 1015 Lausanne, Switzerland
[4]Department of Cell Biology, Harvard Medical School, Boston, MA 02115, USA
[5]Biozentrum, University of Basel, 4056 Basel, Switzerland
[6]Centre for Translational Microbiome Research, Department of Microbiology, Tumor and Cell Biology, Karolinska Institutet, Stockholm 17165, Sweden
[7]Institute Pasteur, Paris, France
[8]ThermoFisher Scientific, Bremen, Germany
[9]Department of Pharmacological & Technological Chemistry, I.M. Sechenov First Moscow State Medical University, Moscow, Russia
[10]The National Medical Research Center for Endocrinology, 115478 Moscow, Russia

*Correspondence and requests for materials should be addressed to R.A.Z. (email: Roman.Zubarev@ki.se)

Contents

Tables......3

Supplementary Figures......4

Tables

Table S1. Comparison of top-down LC-MS/MS analysis of *E. Coli* proteome with normal isotopic composition (full isotope) and isotopically depleted (monoisotopic).

		**Full Isotope spectra**			**Monoisotopic spectra - CHARDA**	
**Proteoform**	**Mono-isotopic Mass, Da**	**Clusters**	**Nr. of fragments**	**Sequence coverage**	**Nr. of fragments**	**Sequence coverage**
**PFR244733: Cold shock-like protein CspC**	7266.72	393	38	39%	199	91%
**PFR244781: DNA-binding protein HU-alpha**	9529.19	494	75	54%	197	81%
**PFR279339: Protein YciI**	10595.42	211	24	20%	133	67%
**PFR244833: DNA-binding protein HU-beta**	9219.99	525	110	54%	295	86%
**PFR245160: 50S ribosomal protein L7/L12**	12198.48	448	74	40%	296	82%

Supplementary Figures

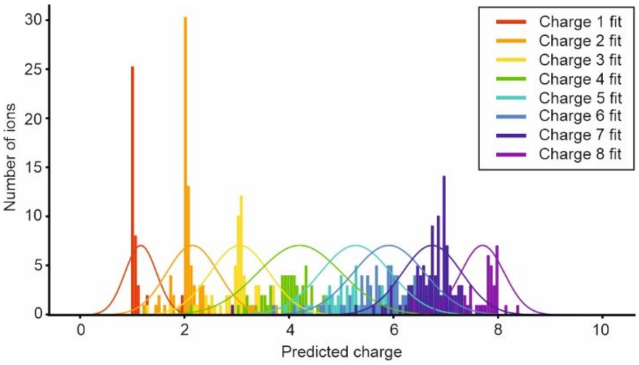

Figure S1. The CHARDA (TDA + MDI) model based on ubiquitin 11+ MS/MS data; the charge resolution is 6.0.

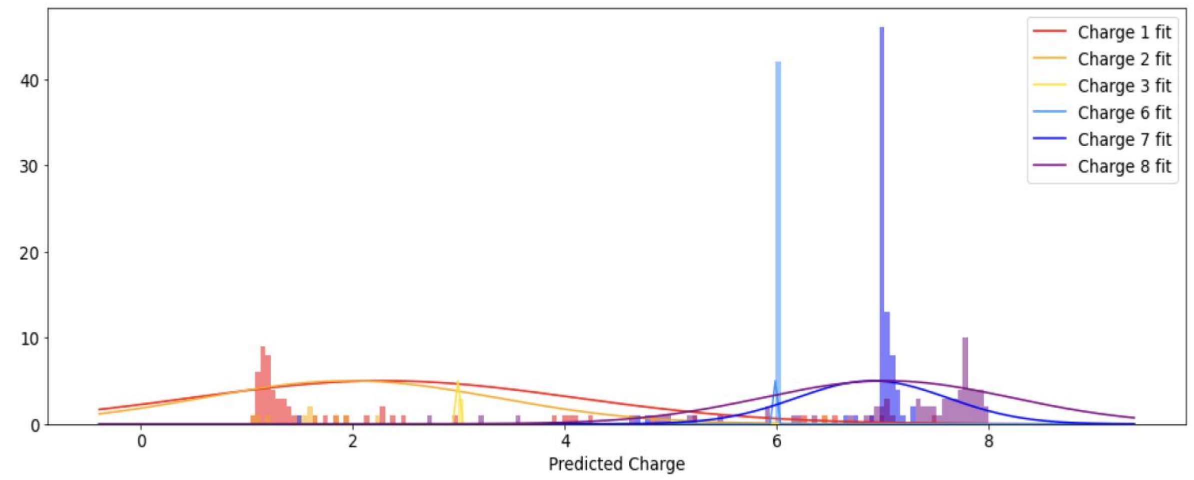

Figure. S2. CHARDA of an MS/MS spectrum of monoclonal antibody Infliximab.

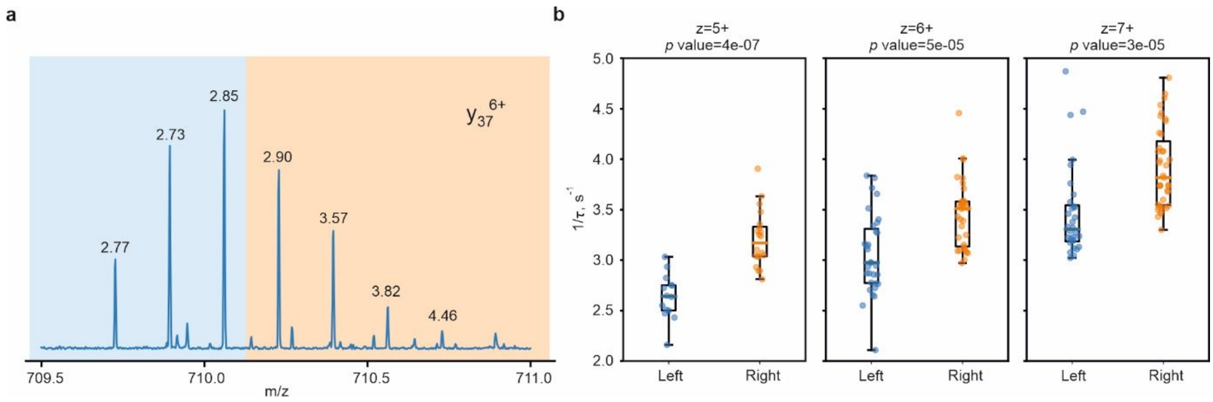

Figure S3. **a**, An example of splitting the isotopic distribution of the y_{37}^{6+} ubiquitin ions into the left and right halves; **b**, decay rate comparison for the left and right halves of the isotopic distributions of ions with *z*=5+, 6+ and 7+. Two-sided Student's t-test for equal variance samples (Center line, median; box limits contain 50%; upper and lower quartiles, 75 and 25%; maximum, greatest value excluding outliers; minimum, least value excluding outliers; outliers, more than 1.5 times of upper and lower quartiles).

a

b

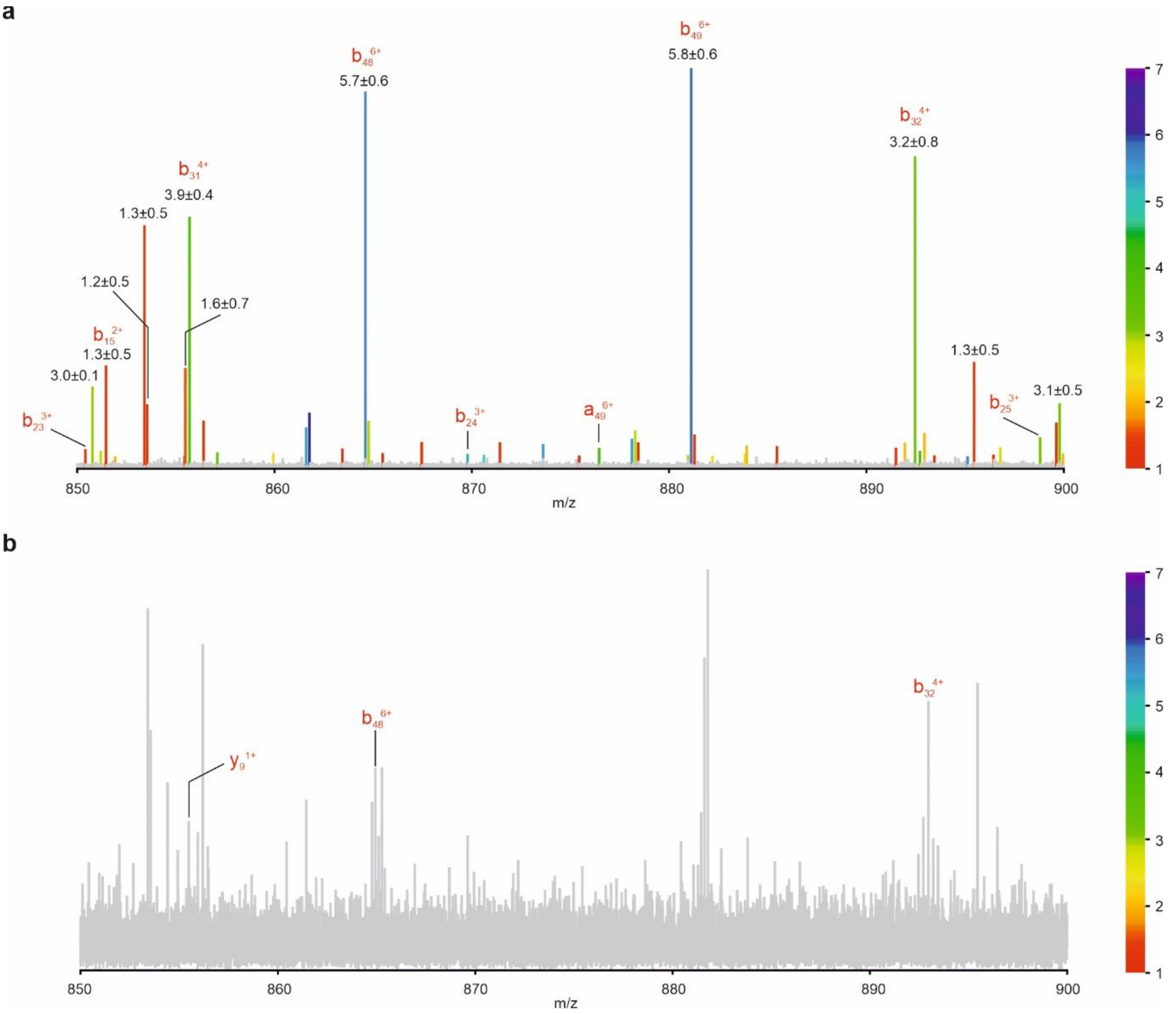

Figure S4. Comparison of the same part of the MS/MS spectrum of *E. Coli* cold shock-like protein CspC from (**a**) the monoisotopic proteome and (**b**) the normal (full-isotope) proteome.
